# Appendiceal Intussusception Secondary to Mantle Cell Lymphoma: A Report of a Rare Case

**DOI:** 10.7759/cureus.97918

**Published:** 2025-11-27

**Authors:** Nariyoshi Miyata, Francis Asomah

**Affiliations:** 1 Department of Surgery, Mount Isa Hospital, Mount Isa, AUS

**Keywords:** appendiceal intussusception, appendiceal neoplasm, gastrointestinal non-hodgkin lymphoma, laparoscopic appendicectomy, laparoscopic right hemicolectomy, mantle cell lymphoma (mcl)

## Abstract

Mantle cell lymphoma (MCL) is a rare, aggressive B-cell non-Hodgkin lymphoma that can involve the gastrointestinal (GI) tract. Appendiceal involvement, however, is highly uncommon. Appendiceal intussusception is a rare entity, accounting for only a small fraction of appendicectomy findings. We present a rare case of a 68-year-old male who developed appendiceal intussusception secondary to mantle cell lymphoma, diagnosed following laparoscopic right hemicolectomy. To the best of our knowledge, this is the first reported case of MCL manifesting with appendiceal intussusception. This report emphasises the significance of considering lymphoproliferative disorders in elderly patients presenting with persistent right lower quadrant pain and imaging suggestive of intussusception.

## Introduction

Mantle cell lymphoma (MCL) accounts for approximately 5-7% of non-Hodgkin lymphomas and is frequently characterised by an aggressive clinical progression [1-3]. It originates from naive B-cells within the mantle zone and is commonly associated with cyclin D1 overexpression due to the t(11;14)(q13;q32) translocation [3-5]. The gastrointestinal (GI) tract is a common extranodal site, identified in up to 30% of cases via endoscopic biopsy; however, presentation within the appendix is exceedingly rare [6-8]. Appendiceal intussusception is an infrequent occurrence, observed in only 0.01% of patients undergoing appendicectomy [9-11]. It may be caused by benign or malignant lesions serving as a lead point. While inflammation and endometriosis are more frequently implicated [12,13], MCL as an etiological factor has not been previously documented. To the authors' knowledge, based on a comprehensive review of the existing literature, this report presents the first known case of appendiceal intussusception attributable to mantle cell lymphoma.

## Case presentation

A 68-year-old male presented to the emergency department with a three-week history of intermittent, dull, colicky right lower quadrant abdominal pain. He denied nausea, vomiting, diarrhoea, or melena but reported a decrease in oral intake and mild constipation. He had no fever or systemic symptoms apart from longstanding fatigue and unintentional weight loss over several years. His medical history was notable for a colonoscopy performed eight months prior for altered bowel habits, which revealed a normal terminal ileum, caecum, and appendiceal orifice, with excellent bowel preparation. His family history included bowel cancer in both his father and sister. He was an ex-smoker, abstinent from alcohol, and had no prior abdominal surgeries.

**Figure 1:**
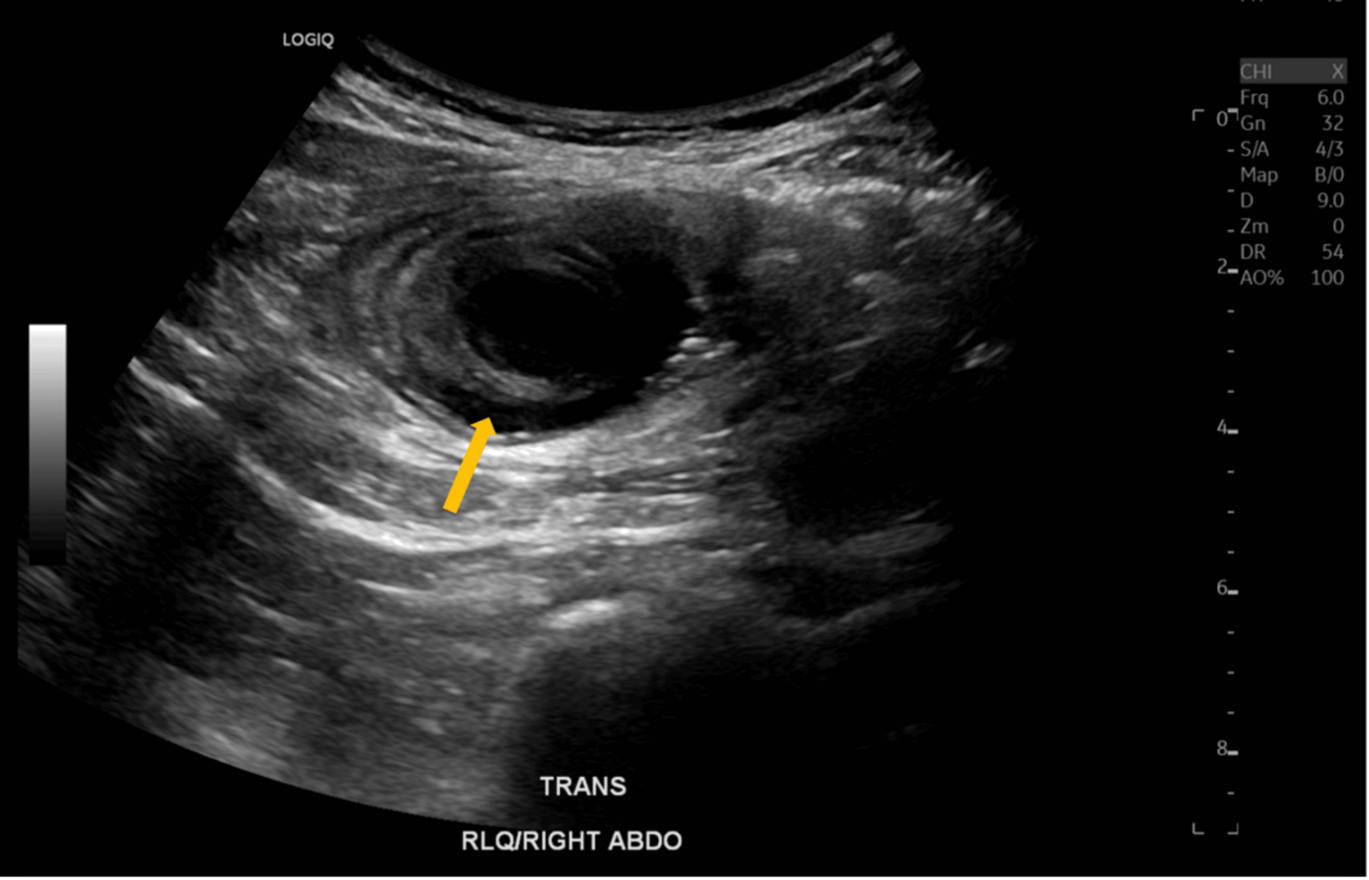
Initial abdominal ultrasound on presentation An arrow indicates intestinal intussusception in the right lower quadrant.

**Figure 2:**
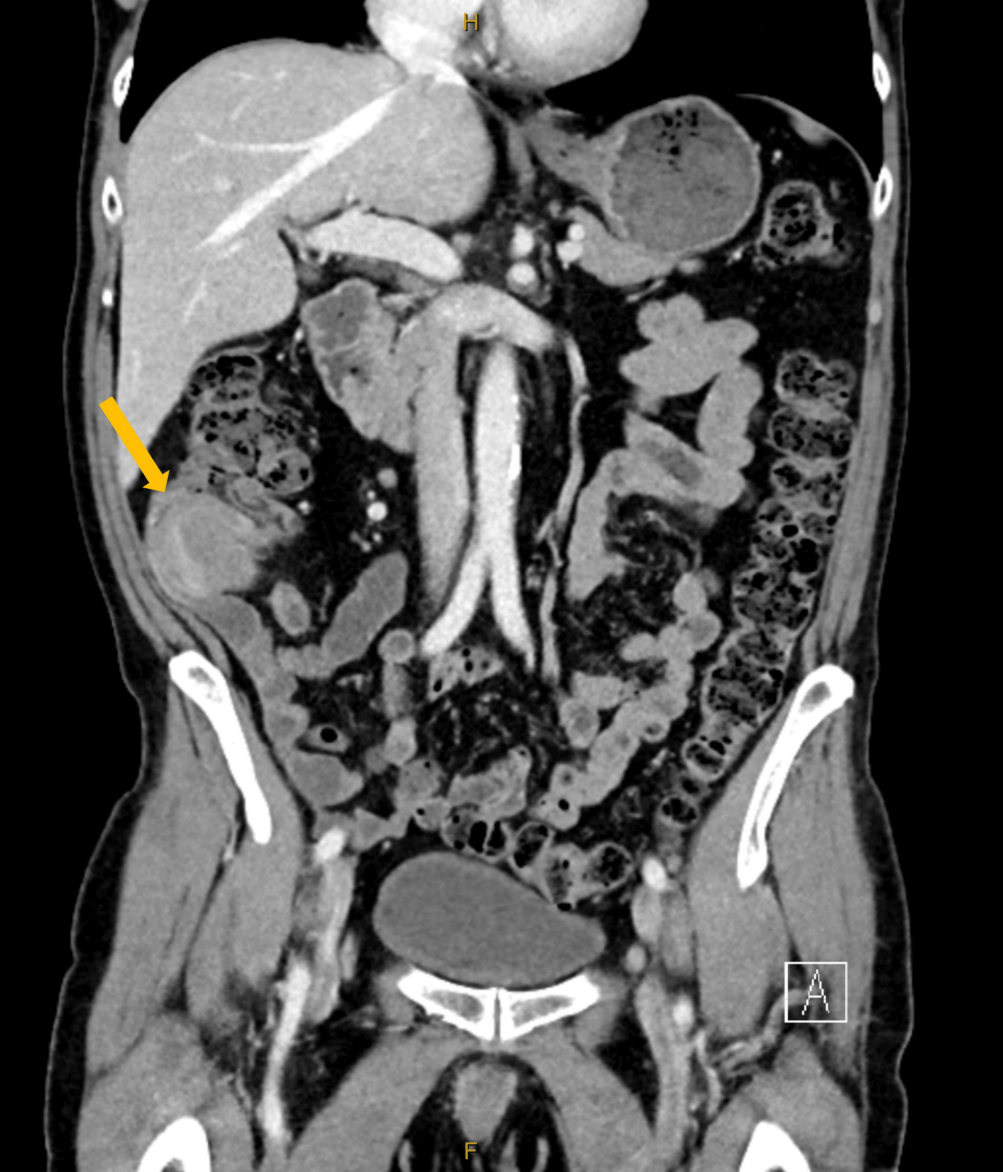
Initial computed tomography (CT) on presentation (coronal view) An arrow indicates intestinal intussusception in the right lower quadrant.

On examination, the patient was found to be haemodynamically stable, afebrile, and exhibiting no signs of acute distress. Abdominal examination revealed tenderness in the right iliac fossa without guarding, rebound tenderness, or palpable mass. Blood tests indicated normal haemoglobin levels, normal white cell count and differential, and normal biochemistry profiles. An abdominal ultrasound demonstrated a target sign in the right lower quadrant, indicative of intussusception (Figure 1). Computed tomography (CT) of the abdomen and pelvis reported as intussusception at the ileocecal junction (Figure 2). The patient was subsequently admitted to the surgical unit and underwent a laparoscopic-assisted right hemicolectomy.

**Figure 3:**
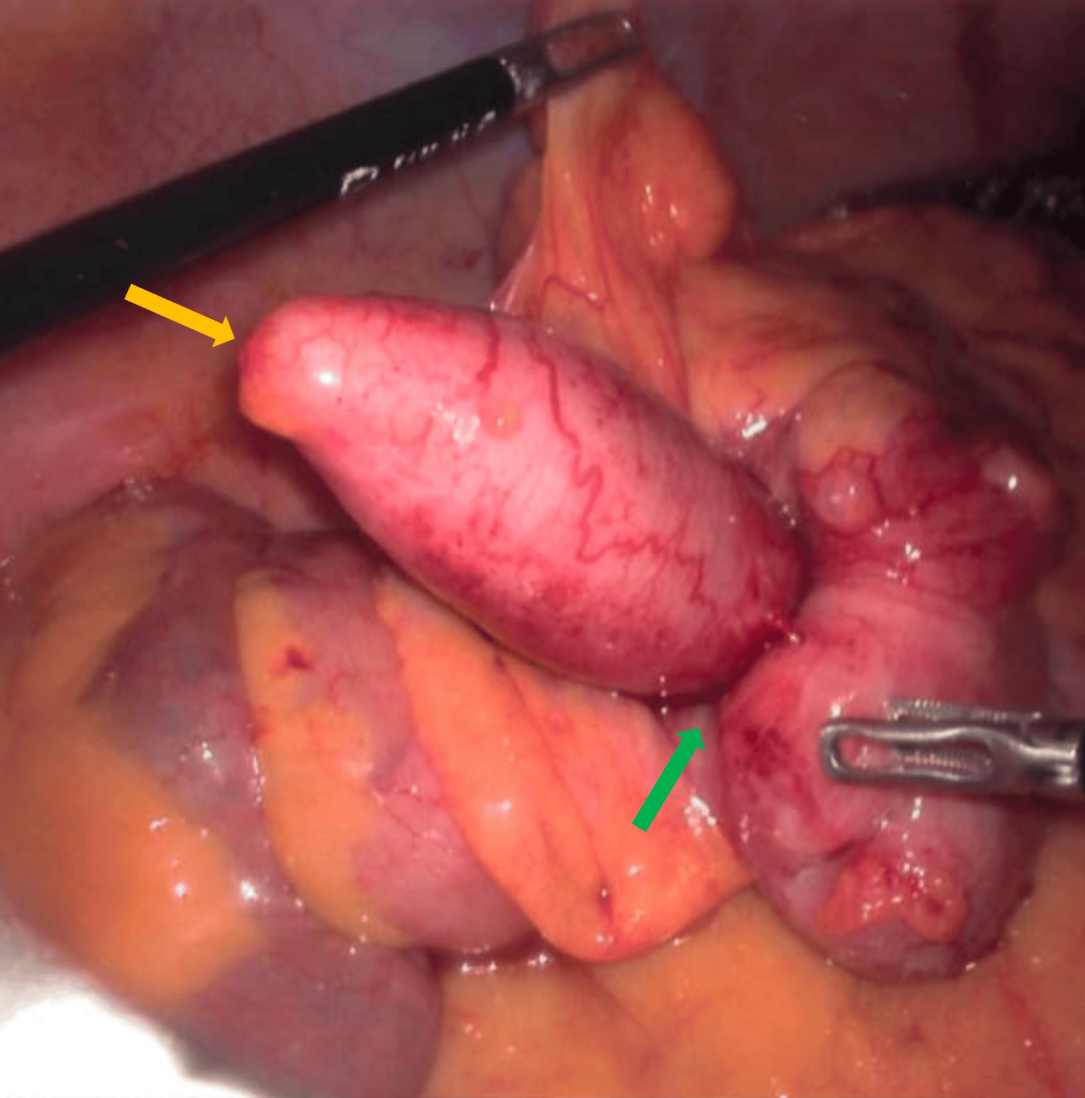
Laparoscopic view of appendiceal intussusception The yellow arrow indicates the appendix, and the green arrow indicates the appendiceal intussusception into the caecum.

**Figure 4:**
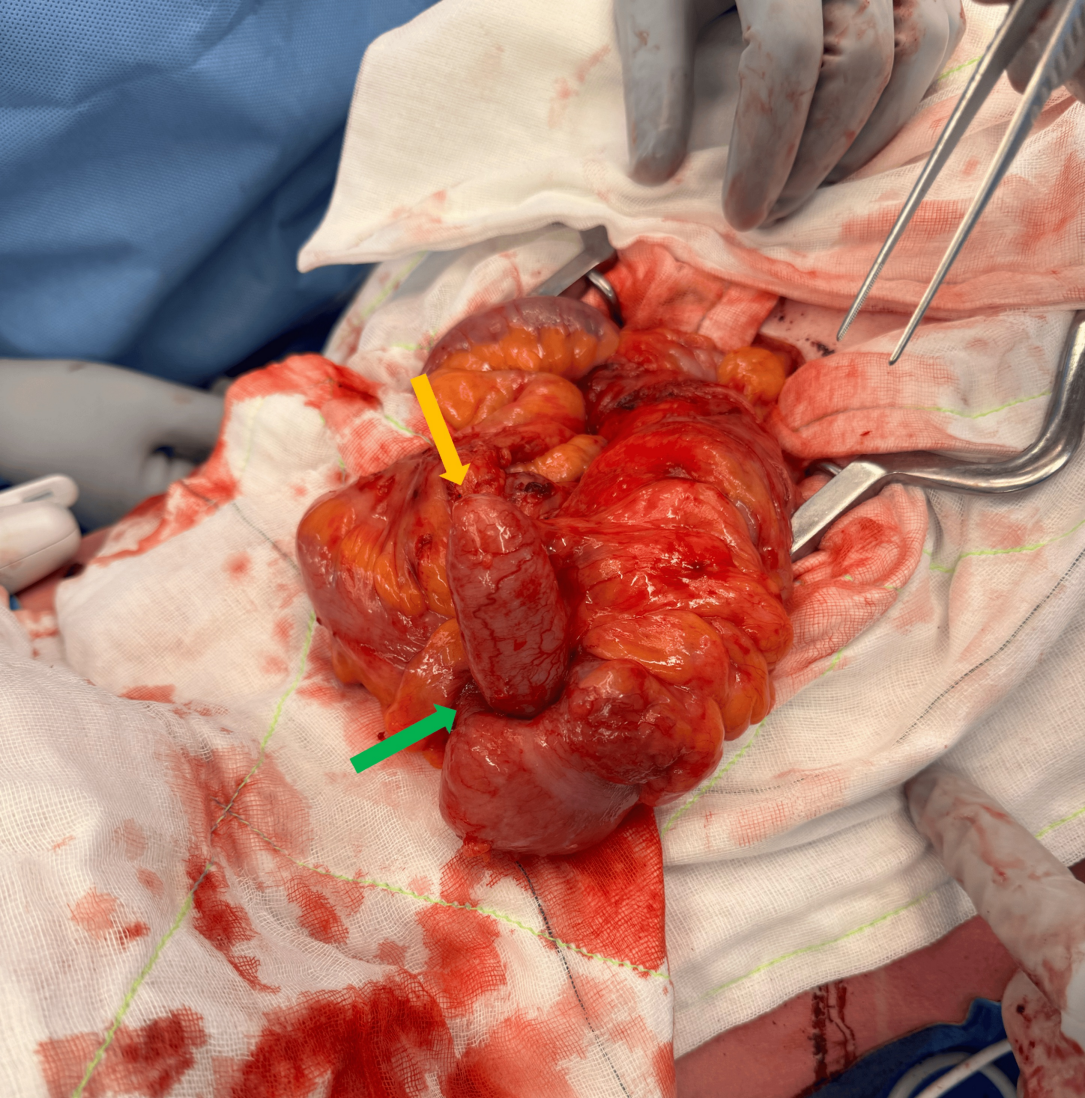
Intraoperative view of appendiceal intussusception The yellow arrow indicates the appendix, and the green arrow indicates the appendiceal intussusception into the caecum.

**Figure 5:**
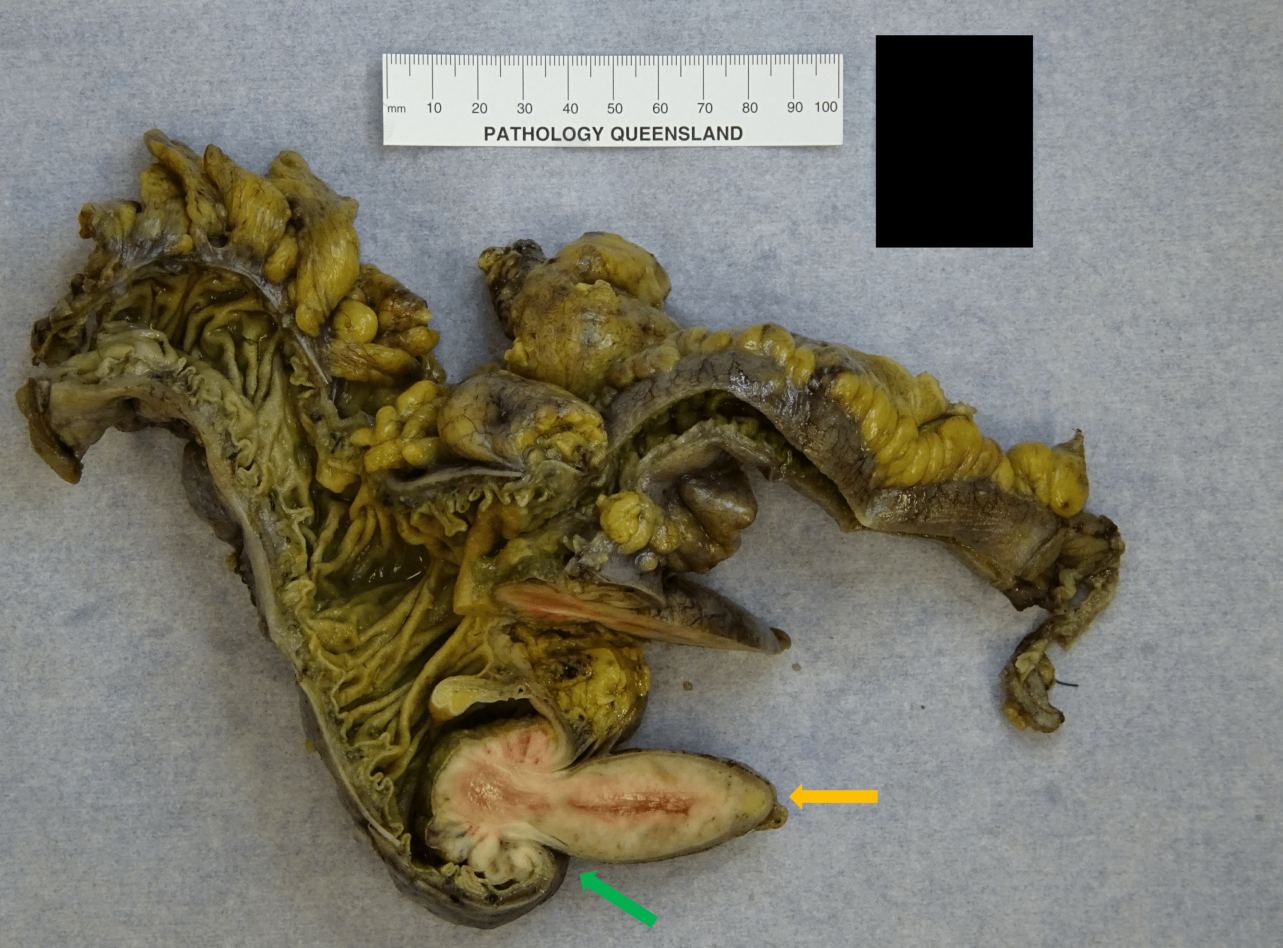
Gross pathology specimen of appendiceal intussusception The yellow arrow indicates the appendix, and the green arrow indicates the appendiceal intussusception into the caecum.

Intraoperative findings revealed a significantly dilated appendix that had undergone intussusception into the caecum (Figures 3-5). There was no evidence of ileocolic intussusception, free mucus, or peritoneal dissemination. The rest of the bowel appeared normal. The postoperative course was uneventful.

**Figure 6:**
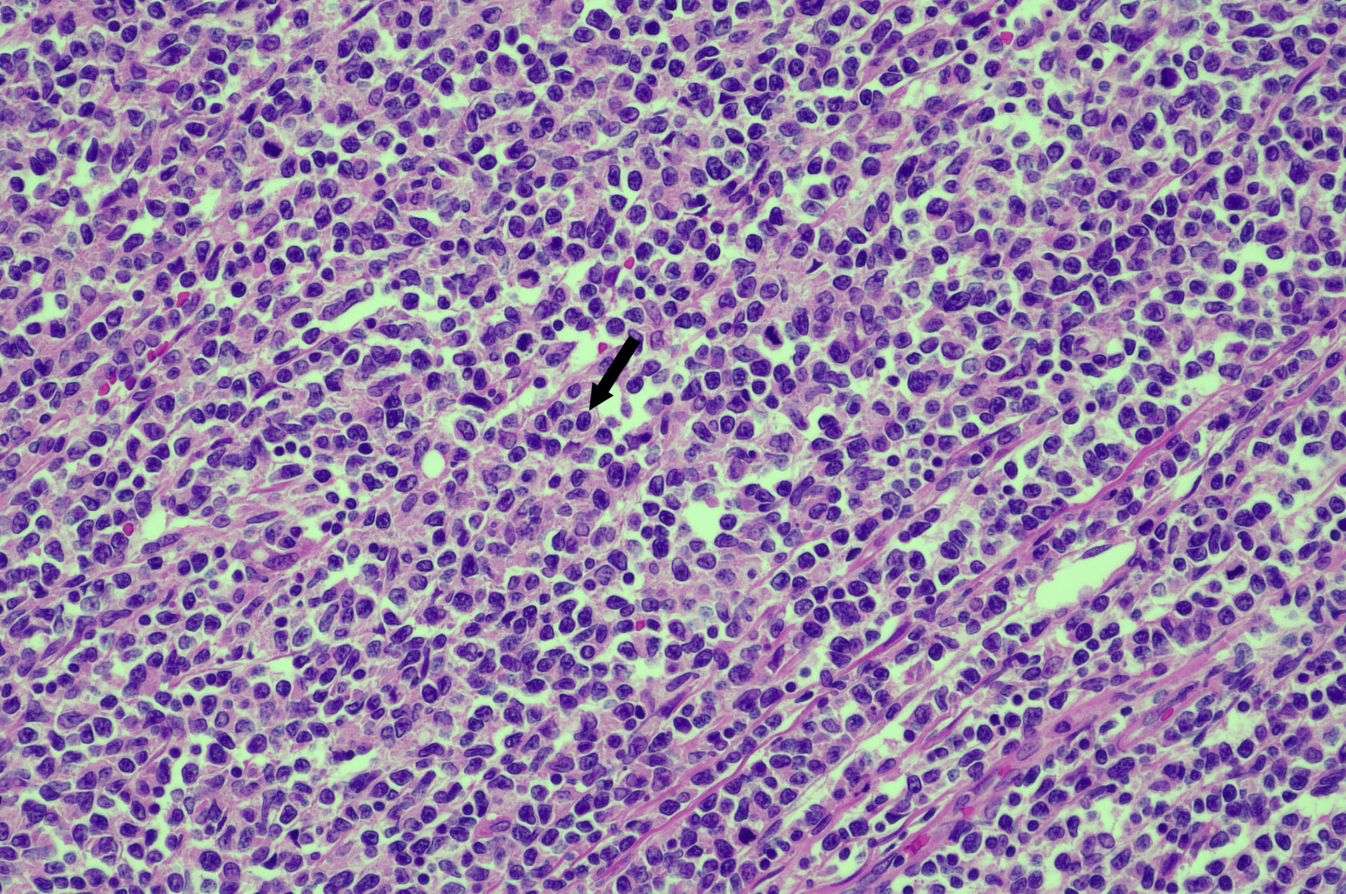
Histopathology of the appendix Specimen showing atypical lymphocytes with moderate nuclear pleomorphism (the arrow indicates one of many atypical lymphocytes).

Histopathology revealed a dense infiltrate of intermediate to large, atypical lymphocytes involving the entire appendix and extending into the caecum (Figure 6). The neoplastic lymphoid infiltrate had a pushing border and obliterated the mucosa and submucosa. There was no evidence of invasion into the serosa or mesenteric fat. A total of 28 mesenteric lymph nodes were sampled, with all demonstrating involvement by mantle cell lymphoma, including the apical node.

**Figure 7:**
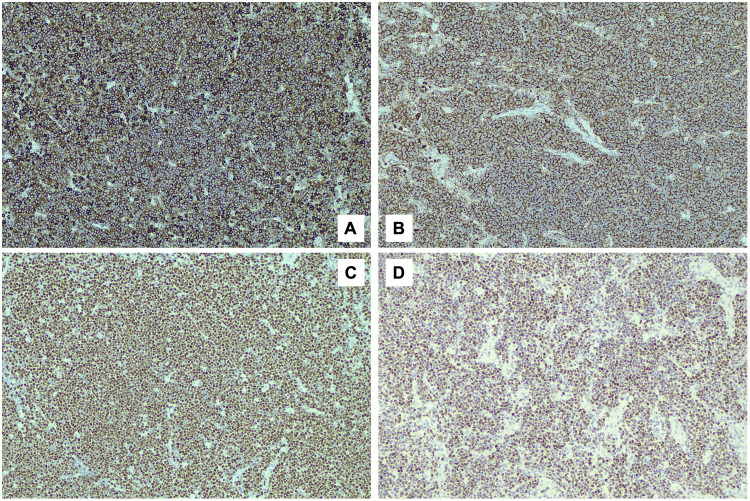
Immunohistochemistry of the appendix A: CD5; B: CD20; C: Cyclin D1; D: SOX11

Immunohistochemistry demonstrated positivity for CD5, CD20, Bcl2, Cyclin D1, and SOX11, while it showed negativity for CD3, CD10, Bcl6, CD23, and EBER (Figure 7). The Ki-67 proliferation index was between 40% and 50%, and p53 exhibited strong diffuse positivity, consistent with a pleomorphic variant of MCL.

**Figure 8:**
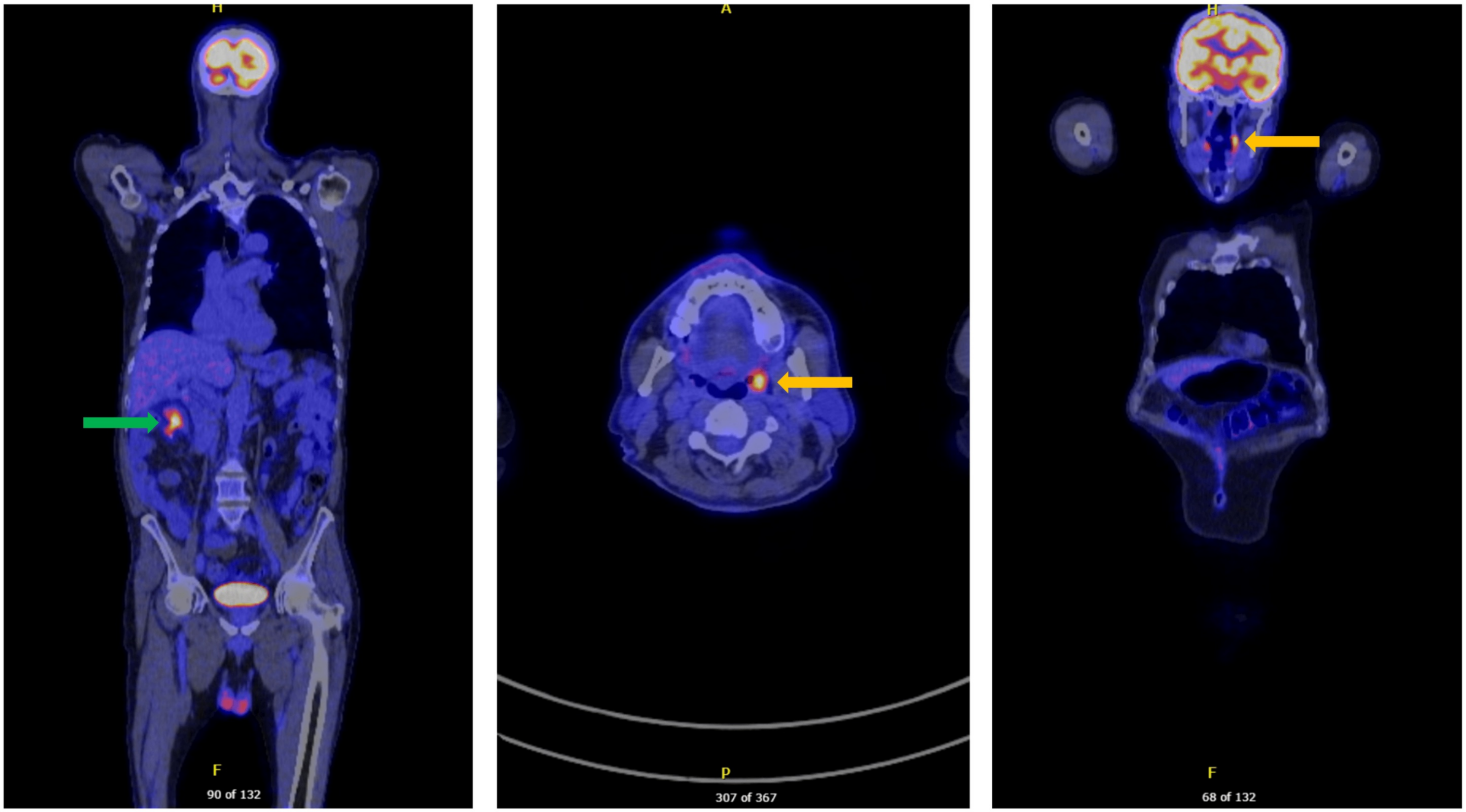
Post-operative initial PET-CT The yellow arrow indicates fluorodeoxyglucose (FDG) uptake in the left tonsil, while the green arrow indicates physiological FDG uptake in the colon. PET-CT: Positron emission tomography-computed tomography.

Postoperatively, the patient underwent staging with positron emission tomography-computed tomography (PET-CT), which revealed asymmetrical fluorodeoxyglucose (FDG) uptake in the left tonsil and physiological uptake in the colon (Figure 8). He was commenced on R-DHAOx (rituximab, dexamethasone, cytarabine, oxaliplatin) and completed four cycles, followed by maintenance rituximab.

**Figure 9:**
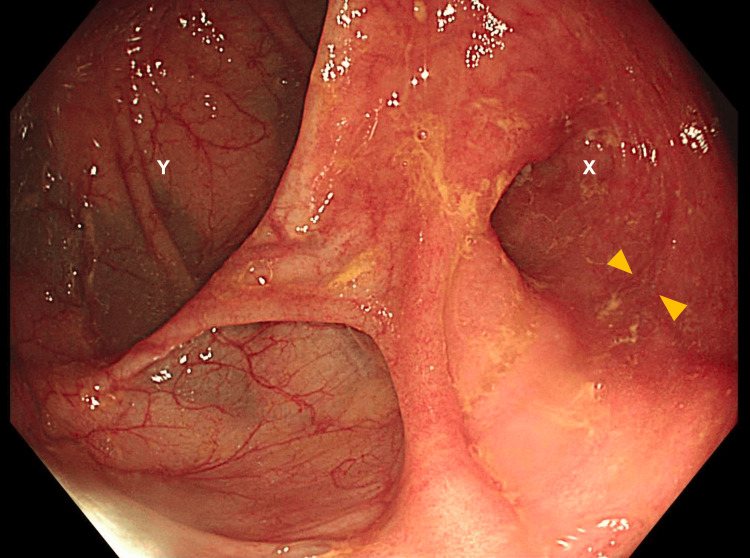
Healthy Ileocolic anastomosis at 15 months post-operative surveillance colonoscopy X: Ileum; Y: Colon; The arrows indicate a healthy ileocolic anastomosis.

**Figure 10:**
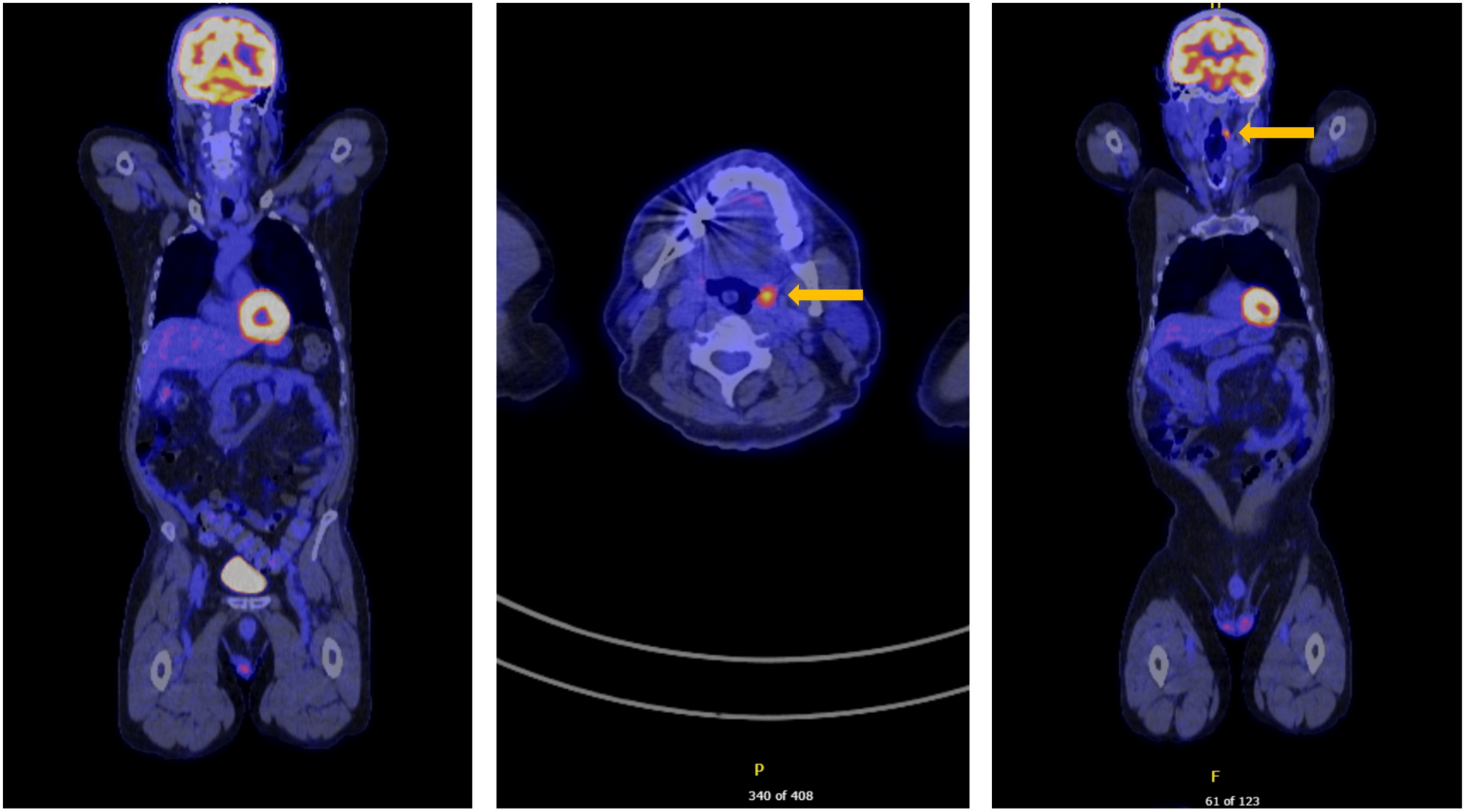
18-month follow-up PET-CT The yellow arrow indicates persistent fluorodeoxyglucose (FDG) uptake in the left tonsil. PET-CT: Positron emission tomography-computed tomography.

Surveillance colonoscopy at 15 months after surgery revealed a healthy ileocolic anastomosis with no signs of recurrence (Figure 9). An 18-month PET scan demonstrated persistent uptake in the left tonsil but no other abnormalities (Figure 10). The patient remains clinically well and in remission.

## Discussion

MCL is a rare and clinically aggressive lymphoma subtype with heterogeneous presentation [1-3]. Gastrointestinal (GI) involvement, while relatively common, is rarely symptomatic and even more infrequently detected in the appendix [6-8]. Studies have demonstrated that GI manifestations can range from subtle mucosal abnormalities to frank mass lesions, often mimicking other pathologies such as adenocarcinoma or Crohn's disease [14,15].

Appendiceal intussusception, frequently presenting as right lower quadrant pain, can be attributable to a lead point such as mucoceles, polyps, or neoplasms. Park et al. described a case involving an appendiceal mucocele causing intussusception that was successfully reduced via colonoscopy [10]. Endometriosis has also been documented as an aetiology in females, as highlighted by Trefois and Coche [12]. Malignant causes are rare, with lymphoma being infrequently implicated [13].

Our case is notable for its uniqueness; mantle cell lymphoma acting as a lead point for intussusception has not been previously documented. A similar case involving MCL and the appendix was reported by Mamukadze et al., where the patient presented with volvulus rather than intussusception [16]. Lee et al. described MCL mimicking acute appendicitis, emphasising the diagnostic challenges [17]. Radiologically, the target sign observed on computed tomography (CT) or ultrasound is characteristic of intussusception [18]. However, preoperative identification of the underlying pathology remains difficult. As demonstrated in our case, a normal colonoscopy performed eight months prior may not preclude rapidly evolving appendiceal pathology.

Histopathological confirmation remains the gold standard, with immunohistochemistry being critical for diagnosis. MCL typically expresses CD5, CD20, Cyclin D1, and SOX11 [3-5]. A high Ki-67 index and strong p53 expression, as observed in our patient, suggest a pleomorphic or aggressive variant, which correlates with a poorer prognosis [19,20]. Therapeutic strategies for MCL vary based on stage and presentation. Surgical resection is essential for localised disease or complications such as intussusception. Systemic chemotherapy, including rituximab-based regimens (e.g., R-CHOP, R-DHAOx), is standard. Maintenance rituximab has demonstrated survival benefits in responders [3]. Long-term surveillance is crucial due to the risk of relapse. PET-CT remains the imaging modality of choice for post-treatment monitoring, as metabolic activity often predates anatomical changes [21].

## Conclusions

This case represents the first report of appendiceal intussusception secondary to mantle cell lymphoma. It emphasises the diagnostic difficulty associated with uncommon aetiologies of abdominal pain and underscores the significance of histopathological evaluation in resected specimens. Clinicians should maintain a heightened suspicion of lymphoma in elderly individuals presenting with atypical gastrointestinal symptoms and intussusception.
